# TLR4 Accessory Molecule RP105 (CD180) Regulates Monocyte-Driven Arteriogenesis in a Murine Hind Limb Ischemia Model

**DOI:** 10.1371/journal.pone.0099882

**Published:** 2014-06-19

**Authors:** Antonius J. N. M. Bastiaansen, Jacco C. Karper, Anouk Wezel, Hetty C. de Boer, Sabine M. J. Welten, Rob C. M. de Jong, Erna A. B. Peters, Margreet R. de Vries, Annemarie M. van Oeveren-Rietdijk, Anton Jan van Zonneveld, Jaap F. Hamming, A. Yaël Nossent, Paul H. A. Quax

**Affiliations:** 1 Department of Surgery, Leiden University Medical Center, Leiden, the Netherlands; 2 Einthoven Laboratory for Experimental Vascular Medicine, Leiden University Medical Center, Leiden, the Netherlands; 3 Department of Biopharmaceutics, Leiden Academic Centre for Drug Research, Leiden University, Leiden, the Netherlands; 4 Department of Nephrology, Leiden University Medical Center, Leiden, the Netherlands; University of Bristol, United Kingdom

## Abstract

**Aims:**

We investigated the role of the TLR4-accessory molecule RP105 (CD180) in post-ischemic neovascularization, i.e. arteriogenesis and angiogenesis. TLR4-mediated activation of pro-inflammatory Ly6C^hi^ monocytes is crucial for effective neovascularization. Immunohistochemical analyses revealed that RP105^+^ monocytes are present in the perivascular space of remodeling collateral arterioles. As RP105 inhibits TLR4 signaling, we hypothesized that RP105 deficiency would lead to an unrestrained TLR4-mediated inflammatory response and hence to enhanced blood flow recovery after ischemia.

**Methods and Results:**

RP105^−/−^ and wild type (WT) mice were subjected to hind limb ischemia and blood flow recovery was followed by Laser Doppler Perfusion Imaging. Surprisingly, we found that blood flow recovery was severely impaired in RP105^−/−^ mice. Immunohistochemistry showed that arteriogenesis was reduced in these mice compared to the WT. However, both in vivo and ex vivo analyses showed that circulatory pro-arteriogenic Ly6C^hi^ monocytes were more readily activated in RP105^−/−^ mice. FACS analyses showed that Ly6C^hi^ monocytes became activated and migrated to the affected muscle tissues in WT mice following induction of hind limb ischemia. Although Ly6C^hi^ monocytes were readily activated in RP105^−/−^ mice, migration into the ischemic tissues was hampered and instead, Ly6C^hi^ monocytes accumulated in their storage compartments, bone marrow and spleen, in RP105^−/−^ mice.

**Conclusions:**

RP105 deficiency results in an unrestrained inflammatory response and monocyte over-activation, most likely due to the lack of TLR4 regulation. Inappropriate, premature systemic activation of pro-inflammatory Ly6C^hi^ monocytes results in reduced infiltration of Ly6C^hi^ monocytes in ischemic tissues and in impaired blood flow recovery.

## Introduction

Arteriogenesis, expansive remodeling of pre-existing collateral arterioles, restores blood flow to tissues distal to an occlusion of a major artery [1]. Arteriogenesis is an inflammation-driven process [2], involving both the adaptive immune system (CD4^+^ T cells [3] [4], CD8^+^ T cells [5], regulatory T cells [6], [7]) and the innate immune system (monocytes [8]–[12], Toll-like receptors).

Toll-like receptors (TLRs) respond to microbial ligands during infection, but also elicit an inflammatory response in reaction to endogenous ligands [13], suggesting that the role of innate immunity exceeds the task of signaling non-self molecules alone. These endogenous ligands are released under sterile conditions as a result of tissue injury, for example after ischemia. In this perspective, endogenous TLR ligands, including Extra Domain A of alternatively spliced fibronectin (EDA), Heat Shock Protein 60 (Hsp60) and High-Mobility Group Box-1 (HMGB1) are present and upregulated in areas of developing collateral arteries [12], [14]. Monocytes, key regulators of arteriogenesis, respond to these endogenous ligands via TLR signaling, resulting in monocyte activation and release of pro-arteriogenic mediators. Furthermore, low levels of circulating TLR-ligands trigger CCR2-induced monocyte migration from the bone marrow via upregulation of MCP1 on bone marrow mesenchymal stem cells [15].

Activation of TLR4, which is expressed on the cell membrane of monocytes and macrophages results in NF-κB mediated inflammatory gene transcription and the release of inflammatory cytokines, including the pro-arteriogenic TNFα [16], [17]. The role of TLR4 in cardiovascular remodeling has been demonstrated in various studies [18]–[23] and more recently also in arteriogenesis [12]. TLR4 deficient mice show impaired blood flow recovery after the induction of hind limb ischemia (HLI), which was suggested to be the result of reduced monocyte accumulation in the perivascular space of remodeling collaterals. Moreover, the exogenous TLR4 ligand lipopolysaccharide (LPS) has been shown to stimulate collateral artery formation after HLI by increasing monocyte recruitment to proliferating collateral arteries [16].

With regards to the role of monocytes in arteriogenesis, it is important to realize that at least two different subtypes of monocytes exist. The “pro-inflammatory” Ly6C^hi^ monocytes are rapidly recruited to sites of inflammation and produce high levels of TNFα [24]. The “repair-associated” Ly6C^l^° monocytes are found in inflamed but also in resting tissue [25]. Moreover, impaired recruitment of Ly6C^hi^ monocytes from the bone marrow results in impaired arteriogenesis [26].

To prevent excessive inflammation, TLR signaling is firmly controlled by, amongst others, TLR accessory molecules. Although several negative regulators of TLR4 have been described in literature, including SIGIRR [27], ST2 [28], Triad3A [29] and several other intracellular proteins [30], we chose to investigate RP105 because of its specificity for inhibition of TLR4-mediated inflammatory response [31], [32]. TLR4 accessory molecule RP105 (also known as CD180) is a TLR4 homologue that is expressed by various cell types, including monocytes and macrophages, but which lacks the intracytoplasmic Toll-IL-1 Receptor (TIR) domain of TLR4. Similar to TLR4, whose function depends on co-expression of MD-2, RP105 expression depends on co-expression of MD-1, an MD-2 homologue [33]. The RP105-MD-1 complex binds to the TLR4-MD-2 complex and consequently inhibits binding of TLR4 to its ligands. This way, RP105 acts as a specific inhibitor of the TLR4-mediated inflammatory response [32]. RP105 has been described to be involved in several inflammatory diseases [31], [34]–[36], including arterial restenosis [37], but the role of RP105 in arteriogenesis is still unknown.

In the present study, we investigated the contribution of RP105 to post-ischemic neovascularization in a hind limb ischemia (HLI) model, using wild type C57BL/6 (WT) and RP105 deficient (RP105^−/−^) mice. Considering that inflammatory TLR4-signaling on monocytes is critical for effective arteriogenesis, we hypothesized that increased TLR4-signaling, through the lack of TLR4 inhibition in RP105^−/−^ mice, would enhance arteriogenesis. Surprisingly, we show here that arteriogenesis is impaired in RP105^−/−^ mice, most likely caused by inappropriate overactivation of monocytes, resulting in impaired monocyte recruitment to remodeling collateral arterioles.

## Methods

All animal experiments were approved by the committee on animal welfare of the Leiden University Medical Center and were performed conform the Directive 2010/63/EU of the European Parliament. All procedures were performed under anesthesia and additional pain relief was applied for invasive procedures.

### Surgical Induction of Hind Limb Ischemia

Mice were anesthetized by intraperitoneal (i.p.) injection of midazolam (8 mg/kg, Roche Diagnostics), medetomidine (0.4 mg/kg, Orion) and fentanyl (0.08 mg/kg, Janssen Pharmaceutica). Unilateral hind limb ischemia was induced by electrocoagulation of the left femoral artery proximal to the superficial epigastric arteries alone or combined with electrocoagulation of the distal femoral artery, proximal to the bifurcation of the popliteal and saphenous artery. After surgery, anesthesia was antagonized with flumazenil (0.7 mg/kg, Fresenius Kabi), atipamezole (3.3 mg/kg, Orion) and buprenorfine (0.2 mg/kg, MSD Animal Health) [38].

For in vivo stimulation, purified 1 µg lipopolysaccharide (LPS) from Escherichia coli K-235 (Sigma-Aldrich) was injected i.p. at day 3 after surgery. Plasma was collected 1 h after LPS injection and plasma TNFα (BD Biosciences) and SAA1 (Kamiya Biomedical Company) levels were measured by enzyme-linked immunosorbent assay (ELISA), according to the manufacturers’ protocols.

### Laser Doppler Perfusion Imaging

Laser Doppler perfusion imaging (LDPI) (Moor Instruments) was used to noninvasively acquire consecutive blood flow measurements. Mice were anesthetized with i.p. injection of midazolam (8 mg/kg) and medetomidine (0.4 mg/kg) and measurements were performed with the mice placed in a double-glassed bowl constantly perfused with water at 37°C. The regions of interest analyzed consisted of the feet distal to the base of the first digit. Paw perfusion was expressed as the ratio of ligated over non-ligated paw.

### Pre-existing Collateral Density

Collateral density between the anterior cerebral artery (ACA), middle cerebral artery (MCA), and posterior cerebral artery (PCA) was determined as described [39]–[41]. Briefly, animals were heparinized systemically and anesthetized by intraperitoneal (i.p.) injection of midazolam (8 mg/kg, Roche Diagnostics), medetomidine (0.4 mg/kg, Orion) and fentanyl (0.08 mg/kg, Janssen Pharmaceutica) prior to vascular casting. Maximal dilation was accomplished by cannulation of the thoracic aorta and infusion of sodium-nitroprusside (30 µg/ml) and papaverine (40 µg/ml) in PBS at 100 mmHg for 3 minutes. Yellow Microfil (Flow Tech Inc.) with viscosity adjusted to prevent capillary and venous filling was infused under a stereomicroscope after craniotomy. The dorsal cerebral circulation was fixed with topical application of 4% paraformaldehyde to prevent any reduction in vessel dimensions after Microfil injection. Brains were incubated in Evans Blue (2 µg/ml) for several days to improve contrast for visualization of the vasculature. Digital images were obtained of the dorsal brain surface and processed with ImageJ software (NIH). Collateral density was calculated by determining the total number of pial collaterals between the ACA-MCA, ACA-PCA and MCA-PCA and dividing by the dorsal surface area of the cerebral hemispheres. Areas that sustained damage, were incompletely filled, or were otherwise uncountable were excluded from analysis.

### Real-time Quantitative PCR

The adductor muscle group of WT mice was harvested before (pre-treatment = pt) and on different time points (day 1, 3, 7, 14, 28) after hind limb ischemia, snap-frozen, crushed, using mortar and pestle, and homogenized over a Qiashredder (Qiagen). Total RNA was extracted using RNeasy fibrous tissue minikit (Qiagen) according to manufacturer’s instructions and RNA integrity was checked using the NanoDrop 1000 Spectrophotometer (NanoDrop Technologies) and the 2100 Bioanalyzer (Agilent Technologies).

Total RNA from whole bone marrow and bone marrow derived monocytes was isolated using a standard Trizol-chloroform extraction protocol. RNA concentration, purity and integrity were examined by nanodrop (Nanodrop Technologies).

For real-time quantitative PCR, RNA was reverse transcribed using High Capacity RNA-to-cDNA kit (Applied Biosystems). Quantitative PCR was performed on the ABI 7500 Fast system, using commercially available TaqMan gene expression assays for TLR4, CD180 (RP105), SIGIRR, ST2L, CTSS, MMP9, MMP2, PI3K, RAC1, GAPDH and RPL13A (Applied Biosystems). Expression levels of GAPDH and RPL13A were used for normalization.

### Immunohistochemistry

For harvesting tissues, mice were anesthetized by intraperitoneal (i.p.) injection of midazolam (8 mg/kg, Roche Diagnostics), medetomidine (0.4 mg/kg, Orion) and fentanyl (0.08 mg/kg, Janssen Pharmaceutica). Mice were sacrificed by exsanguination.

The adductor muscle group and gastrocnemius muscle were harvested and snap frozen in liquid nitrogen or fixed in 3.7% paraformaldehyde.

Serial 5-µm-thick paraffin-embedded sections of ligated and non-ligated adductor muscle group (10 days after HLI) were used for histological analyses of collateral artery size. Vessels at the center of the adductor muscle group, stained using anti-smooth muscle α-actin (αSMA) (DAKO), are likely collaterals but may also include arterioles of the opposing tree. Randomly photographed images through the central part of the adductor muscle group were used to quantify the number and lumen diameter of αSMA^+^ vessels using ImageJ software (NIH) (total of 9 images of 3 sections per mouse).

Serial frozen sections (6 µm) of ligated and non-ligated gastrocnemius muscle (10 days after HLI) were fixed in ice-cold acetone and used for histological analyses of capillary density. Sections were stained using anti-CD31 (BD Biosciences). Randomly photographed images through the gastrocnemius muscles were used to quantify the number of CD31^+^ vessels per section using ImageJ software (NIH) (total 6 sections per mouse).

Paraffin-embedded sections of gastrocnemius and adductor muscle group harvested 1 day after HLI were stained for RP105 positive cells with a rabbit anti-RP105 antibody (Abcam).

Frozen sections of adductor muscle group harvested 1 day after HLI (6 µm) were fixed in ice-cold acetone and stained with anti-RP105 (Abcam), Cy3 conjugated anti-αSMA (Sigma-Aldrich) and anti-MOMA-2 (Millipore). MOMA-2 was visualized using an Alexa 488-conjugated secondary antibody (Invitrogen) and RP105 using an APC-conjugated secondary antibody. Nuclei were stained with Vectashield with DAPI (Vector Laboratories). Fluorescent pictures were taken on a LSM700 microscope (Carl Zeiss) and adjusted using Zen 2009 software (Carl Zeiss).

### Ex vivo Whole Blood Stimulation

Blood was collected from the tail vein from WT and RP105^−/−^ mice and diluted 1∶25 in RPMI 1640 (Invitrogen) supplemented with non-essential amino acids (PAA Laboratories) and glutamax (Invitrogen). Samples were incubated overnight at 37°C, 5% CO2, in absence or presence of LPS (0–75 ng) from Escherichia coli K-235 (Sigma-Aldrich). Cell-free supernatant was collected and TNFα and IL6 levels were measured by ELISA according to the manufacturer’s protocol (BD Biosciences).

### Flow Cytometry

For assessment of ex vivo inflammatory response of circulating cells, peripheral blood was drawn from the tail vein of RP105^−/−^ and WT mice. Whole blood was incubated for 3 hours at room temperature, in presence of PBS or 50 ng/ml LPS (Sigma-Aldrich).

For assessment of in vivo inflammatory response, mice were sacrificed before (t0) and 1 day after (t1) hind limb ischemia. Blood was drawn from tail vein. Total circulating leucocytes were measured using the KX-21N Hematology Analyzer (Sysmex). The adductor muscle group, gastrocnemius muscle, spleen and bone marrow were harvested and collected in 3% BSA. Skeletal muscles were first chopped up and incubated for 1 h at 37°C in the presence of 450 U/ml collagenase I (Worthington Biochemical Corporation) and 60 U/ml DNAse I (Sigma-Aldrich). Tissues were minced through 40 µm-cell strainers (BD Biosciences) to obtain single cell suspensions and resuspended in IMDM (Lonza) with 2% FCS. CD45^+^ leukocytes were isolated from the skeletal muscles using magnetic MicroBeads conjugated to rat anti-CD45 antibodies (Miltenyi Biotec), according to manufacturer’s protocol.

Fluorochrome-conjugated antibodies used were anti-mouse CD11b-APC (BD Biosciences), CD115-PerCP (R&D Systems), Ly6C-Alexa 488 (AbD Serotec), Ly6G-PE (BD Biosciences) and B220-APC eFluor780 (eBioscience). Cells were counted on a LSRII flow cytometer (BD Biosciences) and data was analyzed using FACSDiva software (BD v6.1.2). The gating strategy is shown in Figure S3.

### Monocyte Isolation

For harvesting bone marrow, mice were anesthetized by intraperitoneal (i.p.) injection of midazolam (8 mg/kg, Roche Diagnostics), medetomidine (0.4 mg/kg, Orion) and fentanyl (0.08 mg/kg, Janssen Pharmaceutica). Mice were sacrificed by cervical dislocation.

Bone marrow derived monocytes were isolated and differentiated as described previously [42]. In brief, bone marrow suspensions were isolated from WT and RP105^−/−^ mice by flushing the femurs and tibias with PBS. Cells were cultured for 5 days in RPMI medium supplemented with 10% fetal calf serum, 2 mmol/L l-glutamine, 100 U/mL penicillin and 100 µg/mL streptomycin and 20 ng/mL recombinant murine M-CSF (eBioscience) to generate BM-derived monocytes. After 5 days, non-adherent cells were harvested and analyzed by FACS for CD11b and Ly6C expression in order to determine monocyte purity (∼84%).

### Migration Assay

WT and RP105^−/−^ bone marrow-derived monocytes (10^5^ cells per well) were applied in quadruplicate to the upper chamber of a transwell system (24 wells, 8 µm pore size, PAA) in RPMI supplemented with 0.05% BSA (Sigma-Aldrich) and PBS or LPS in PBS (10 ng/ml) was added. Monocyte chemotaxis was assessed towards 100 nM FMLP (formyl-methionyl-leucyl-phenylalanine, Sigma-Aldrich) in the basolateral chamber. After 2 hours of incubation the number of monocytes migrated to the basolateral chamber was counted manually.

### Statistical Analysis

All results are presented as mean ± standard error of the mean (SEM) or as scatter plot. Comparisons between groups were performed using Student’s t-tests. FACS data on tissues from mice before and after induction of hind limb ischemia were analyzed using a one-way ANOVA (Kruskall Wallis statistics) with a Dunn’s multiple comparison test to be able to compare all 4 study groups. P-values<0.05 are indicated by *, p-values<0.01 and <0.001 are indicated by ** and ***, respectively.

## Results

### RP105 Expression in Arteriogenesis

**Figure 1:**
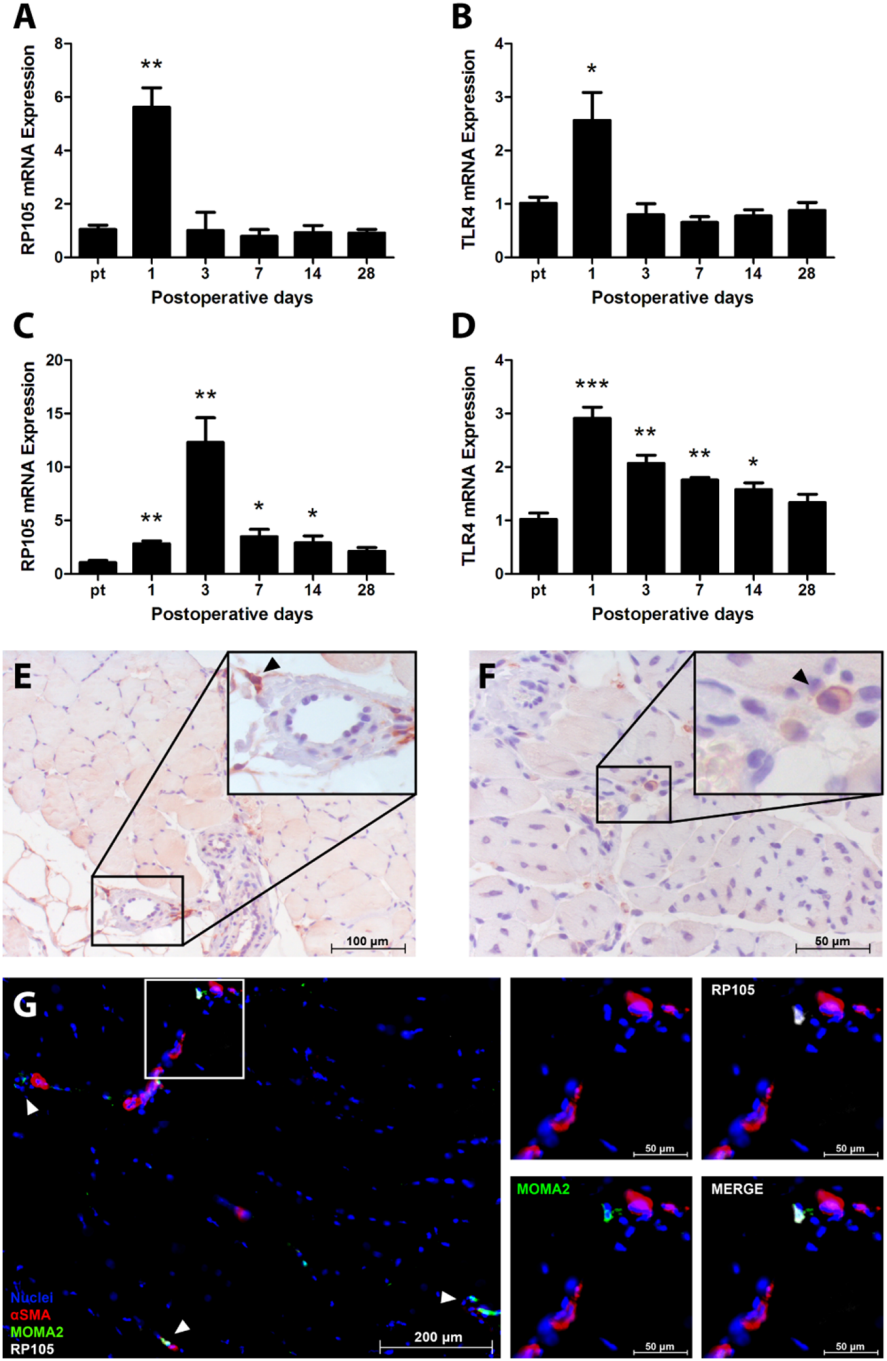
RP105 and TLR4 in arteriogenesis in WT mice. RP105 mRNA (**A**) and TLR4 mRNA (**B**) expression in the adductor muscle group after induction of HLI, measured by real-time quantitative PCR. RP105 mRNA (**C**) and TLR4 mRNA (**D**) expression in the ischemic gastrocnemius muscle after induction of HLI, measured by real-time quantitative PCR (n = 4 mice per time point). Expression levels were normalized against either GAPDH or RPL13A. pt = pre-treatment. All values are presented as the mean ± SEM. *P<0.05, **P<0.01, ***P<0.001, calculated against pre-treatment. Immunohistochemical staining on paraffin-embedded WT adductor muscle group (**E**) and gastrocnemius muscle (**F**) 1 day after induction of HLI, using anti-RP105 antibodies. Black arrowheads denote RP105^+^ cells. (**G**) Immunohistochemical staining on fresh-frozen sections of WT adductor muscle 1 day after induction of HLI, using anti-αSMA (red), anti-RP105 (white) and anti-MOMA-2 (green) antibodies. Cell nuclei were stained with DAPI (blue). White arrowheads denote RP105^+^MOMA-2^+^ cells.

To determine whether RP105 expression is regulated during arteriogenesis, the adductor muscle group containing remodeling collateral arteries of WT mice was harvested before and after (day 1, 3, 7, 14 and 28) induction of HLI [38]. RP105 mRNA levels were significantly increased at day 1 in comparison to baseline levels (Figure 1A, p = 0.001) and mimic TLR4 mRNA expression (Figure 1B, p = 0.03). Both RP105 and TLR4 were transiently upregulated at day 1 and returned to baseline levels by day 3. Also in the ischemic gastrocnemius muscle, RP105 and TLR4 mRNA levels were increased after HLI (Figure 1C–D).

Since RP105 is known to be expressed by circulating cells [32], we performed immunohistochemical staining on sections of paraffin-embedded adductor muscle group and gastrocnemius muscle, harvested 1 day after HLI, to determine whether the influx of RP105^+^ inflammatory cells contributed to the increase in RP105 mRNA levels. As expected, RP105^+^ cells were observed near blood vessels in adductor and gastrocnemius muscle (Figure 1E and 1F respectively).

In immunofluorescently stained fresh-frozen sections of adductor muscle group at 1 day after HLI, RP105^+^ cells in proximity of αSMA^+^ vessels were also positive for MOMA-2, indicating that RP105^+^ cells in this tissue are predominantly monocytes/macrophages (Figure 1G).

### RP105 Contributes to Collateral Remodeling

**Figure 2:**
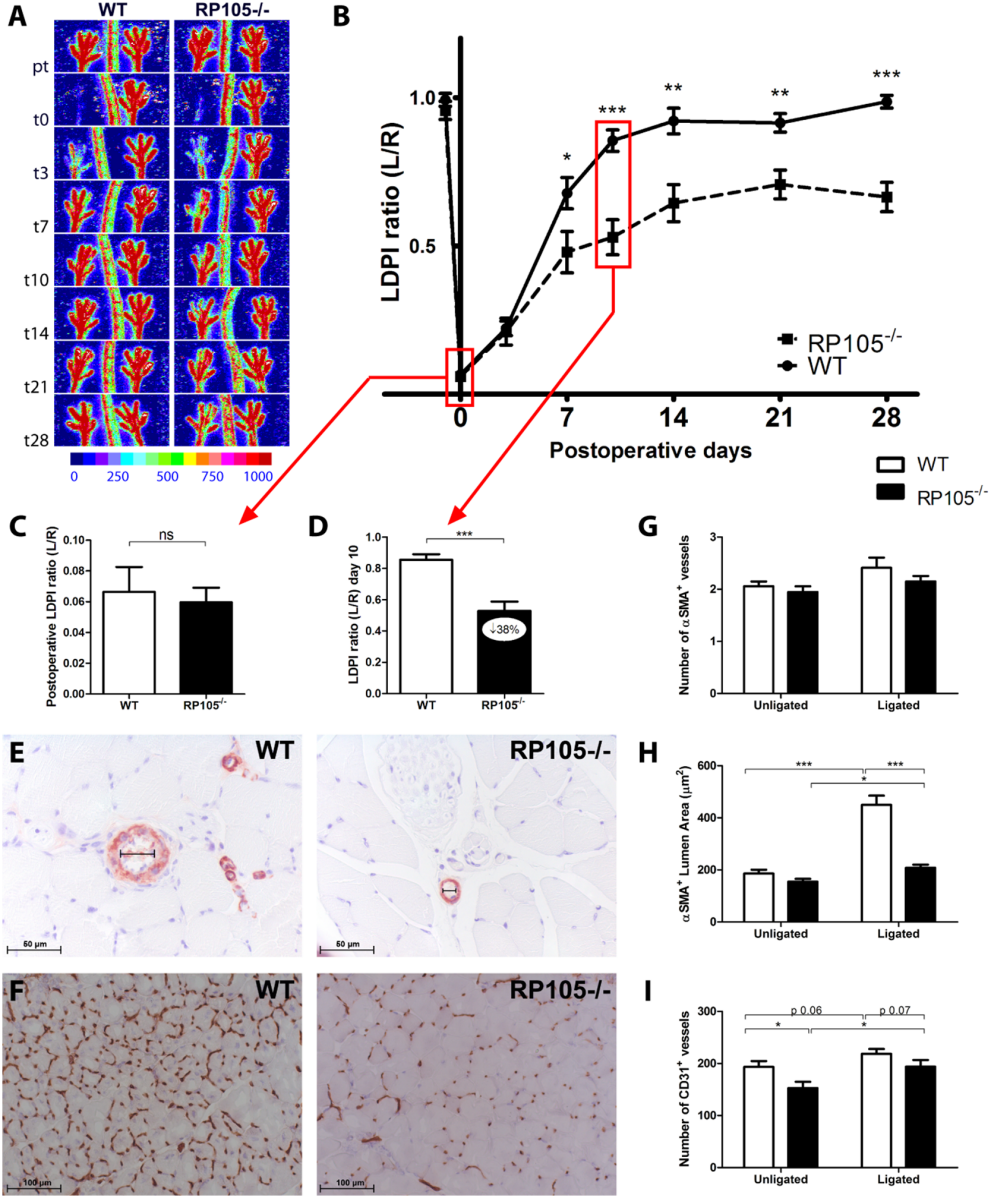
Blood flow recovery in RP105^−/−^ mice. (**A**) Representative Laser Doppler Perfusion Imaging (LDPI) images of paws from WT and RP105^−/−^ mice after induction of HLI in the left limb. High blood flow is displayed in red. (**B**) Quantification of LDPI measurements of RP105^−/−^ (n = 10) and WT (n = 9) mice over time. Data are calculated as the ratio of ligated over non-ligated paw. (**C**) Quantification of LDPI measurements of WT and RP105^−/−^ mice directly after induction of HLI. (**D**) Quantification of LDPI measurements 10 days after induction of HLI. (**E**) Immunohistochemical staining of paraffin-embedded adductor muscle group of WT (n = 6) and RP105^−/−^ (n = 6) mice, 10 days after HLI, using anti-αSMA (red) antibodies. Smallest lumen diameter of αSMA^+^ vessels is indicated by black bars. (**F**) Immunohistochemical staining on fresh frozen sections of gastrocnemius muscles of WT (n = 6) and RP105^−/−^ (n = 6) mice, 10 days after HLI, using anti-CD31 (brown) antibodies. Number (**G**) and lumen area (µm^2^) (**H**) of αSMA^+^ vessels, measured at the center of the adductor muscle group in ligated and non-ligated limbs of RP105^−/−^ and WT mice. (**I**) Capillary density in gastrocnemius muscles, defined as the number of CD31^+^ vessels per section. pt = pre-treatment. ns = non-significant. All values are presented as the mean ± SEM. *P<0.05, **P<0.01, ***P<0.001.

Having determined that RP105 expression is upregulated during collateral remodeling, RP105^−/−^ and WT mice were subjected to HLI [38]. Unexpectedly, blood flow recovery in RP105^−/−^ mice was significantly reduced (Figure 2A–B). Blood flow directly after HLI decreased equally in both groups, to approximately 6% of blood flow in the contralateral limb (Figure 2C). However, recovery in RP105^−/−^ mice was reduced by 38% compared to WT mice 10 days after HLI (Figure 2D, p<0.001).

This reduction in blood flow recovery was confirmed by quantification of α-smooth muscle actin positive (αSMA^+^) vessels in the adductor muscle group (Figure 2E) and CD31^+^ vessels in the ischemic gastrocnemius muscle (Figure 2F), harvested 10 days after HLI.

Although the absolute number of αSMA^+^ vessels in the adductor muscle group were similar (Figure 2G), the expansive remodeling of αSMA^+^ arterioles was significantly reduced in RP105^−/−^ mice compared to WT mice following HLI. In RP105^−/−^ mice, the mean lumen area per αSMA^+^ vessel (RP105^−/−^208±12 µm^2^ vs WT 450±36 µm^2^, p<0.001) (Figure 2H) and the total αSMA^+^ vessel area per section (RP105^−/−^443±19 µm^2^ vs WT 1059±72 µm^2^, p<0.001) (Figure S1A) were reduced by 54% and 58% respectively compared to WT mice. Also, the fraction of larger αSMA^+^ vessels was significantly higher in WT mice (Figure S1B, p<0.05).

RP105^−/−^ mice did not show a significant decrease in angiogenic response, as measured by the capillary density and size in the ischemic gastrocnemius muscle (Figure 2I and Figure S1C). In contrast, the capillary density appeared to increase slightly more in RP105^−/−^ mice after HLI, which would be plausible considering the increased severity of ischemia in the mice due to impaired arteriogenesis compared to WT mice. However, when we compared the index of capillary size and density in the treated over the untreated paws, there were no significant differences between the two strains (Figure S1D–E). The pre-existing capillary density however was slightly lower in RP105^−/−^ mice compared to WT mice (Figure 2I and Figure S1C).

**Figure 3:**
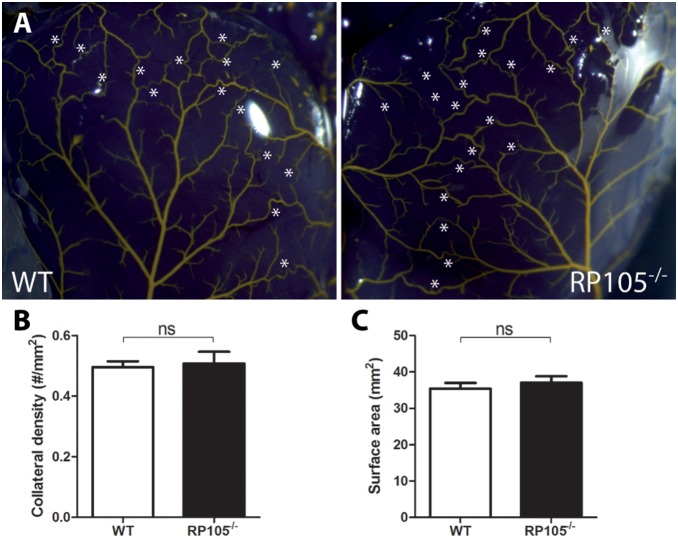
Pre-existing collateral bed in RP105^−/−^ mice. (**A**) Representative images of the pial circulation in WT and RP105^−/−^ mice. White asterisks indicate collateral arteries between anterior, middle and posterior cerebral arteries (ACA, MCA and PCA, respectively). (**B**) Pial collateral density was calculated in WT (n = 4) and RP105^−/−^ (n = 4) mice, dividing the sum of ACA to MCA, ACA to PCA and MCA to PCA connectors by the surface area of the cerebral hemispheres. (**C**) Region of the brain utilized for calculation of pial density. Areas were excluded when they were damaged, had poor filling with Microfil, or were otherwise uncountable. ns = non-significant. All values are presented as the mean ± SEM.

To exclude pre-existing differences in the number of collateral arterioles available for arteriogenesis, collateral density in the pial circulation was assessed in both RP105^−/−^ and WT mice using arterial vascular casting [26], [40]. Pre-existing collateral density in the pial circulation of the dorsal cerebral cortex predicts collateral density in skeletal muscle and other vascular beds [39]–[41]. Pial collateral density was similar between RP105^−/−^ and WT mice (Figure 3A–C), which was in agreement with a similar drop in paw perfusion directly after induction of HLI in both strains (Figure 2C).

### RP105 Inhibits Inflammatory Response

**Figure 4:**
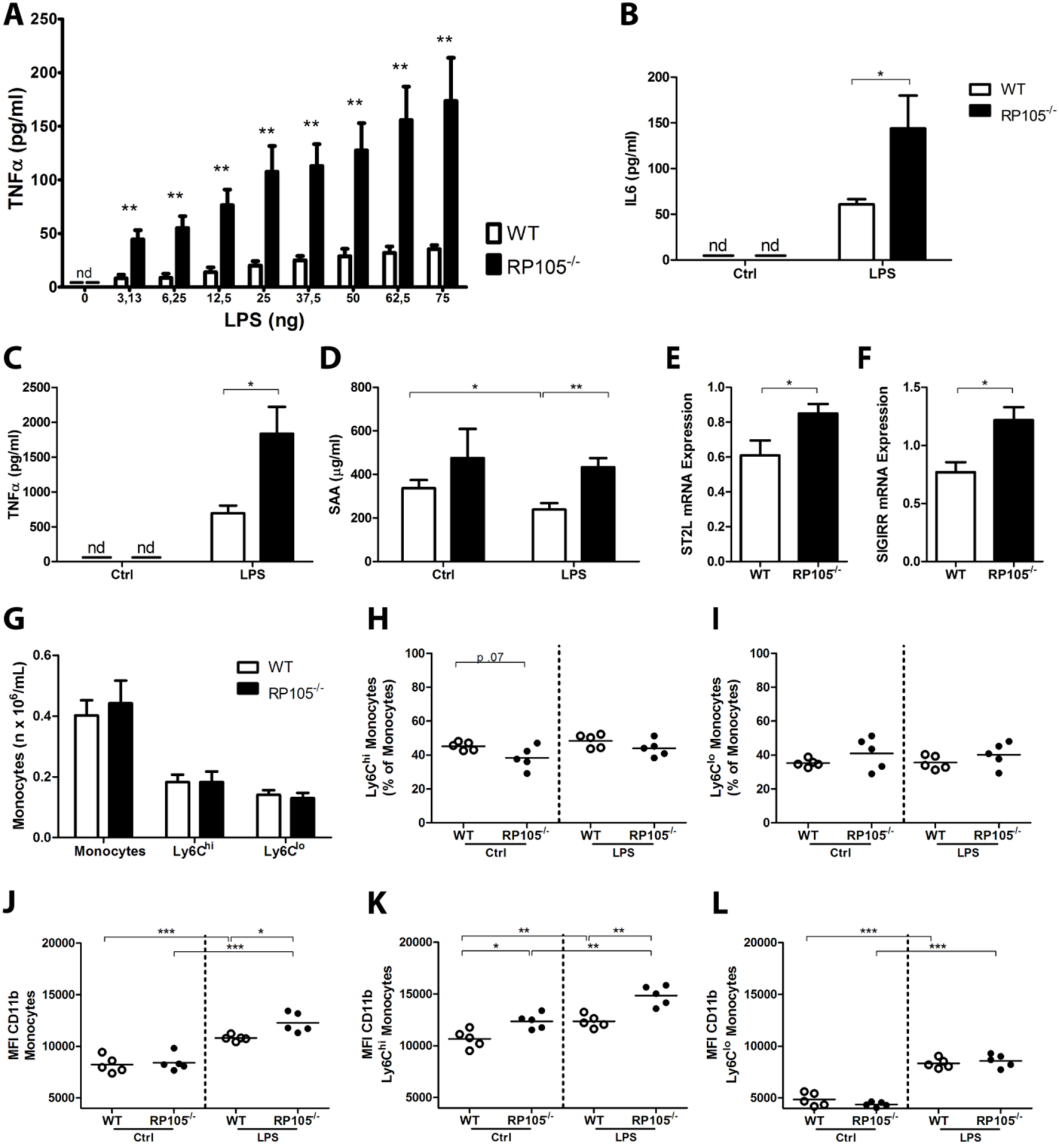
Inflammatory response in RP105^−/−^ mice. Blood from WT and RP105^−/−^ mice was collected, diluted (1∶25) and incubated for 24 h with LPS (0–75 ng) ex vivo. TNFα (pg/ml) (**A**) and IL6 (pg/ml) (**B**) levels in cell-free supernatant were measured by ELISA (n = 5 WT; n = 5 RP105^−/−^). Plasma TNFα levels (pg/ml) (**C**) and SAA1 (µg/ml) (**D**) in RP105^−/−^ and WT mice, 1 h after intraperitoneal injection of LPS (1 µg/mouse) (n = 8 WT PBS; n = 9 WT LPS; n = 9 RP105^−/−^ PBS; n = 10 RP105^−/−^ LPS). ST2L mRNA (**E**) and SIGIRR mRNA (**F**) expression in the adductor muscle group 10 days after induction of HLI, measured by real-time quantitative PCR (n = 6 WT; n = 6 RP105^−/−^). (**G**) Flow cytometry analysis of monocytes and monocyte subtypes (Ly6C^hi^ and Ly6C^l^°) in RP105^−/−^ and WT mice. Values are presented as total counts in blood (n x10^6^/mL). Fraction of Ly6C^hi^ (**H**) and Ly6^l^° (**I**) subtypes of total monocytes in RP105^−/−^ and WT mice after incubation with LPS or control ex vivo. Activation state of total monocytes (**J**), Ly6C^hi^ monocytes (**K**) and Ly6^l^° monocytes (**L**) in whole blood incubated with LPS or control ex vivo, measured by mean fluorescence intensity (MFI) of CD11b (n = 5 WT PBS; n = 5 WT LPS; n = 5 RP105^−/−^ PBS; n = 5 RP105^−/−^ LPS). nd = non-detectable, ctrl = control. All values are presented as the mean ± SEM. *P<0.05, **P<0.01, ***P<0.001.

Since arteriogenesis is an inflammation-driven process and RP105 has been described to regulate TLR4 signaling, we investigated the role of RP105 in inflammatory responses ex vivo and in vivo. LPS stimulation of whole blood from both mouse strains resulted in a dose-dependent increase in TNFα and IL6 production, however this response was drastically higher in blood from RP105^−/−^ mice (Figure 4A and B, p<0.01 for all tested concentrations for TNFα, p<0.05 for IL6). In vivo, intraperitoneal (i.p.) injection of LPS (1 µg) resulted in higher TNFα plasma levels in RP105^−/−^ mice compared to WT mice (Figure 4C, p = 0.02). To monitor for sepsis, plasma Serum Amyloid A1 (SAA1) levels were measured. Although SAA1 levels were higher in RP105^−/−^ mice than in WT mice, levels did not increase further after LPS injection in either strain (Figure 4D). Mice showed no visible signs of sepsis or disease either in response to 1 µg LPS.

### RP105^−/−^ Mice Upregulate other TLR Inhibitors

To determine whether the loss of RP105 expression leads to compensatory mechanisms in RP105^−/−^ mice, we measured expression of other TLR inhibitory molecules in total RNA isolated from the adductor muscles of WT and RP105^−/−^ mice at 10 days after induction of HLI. Expression of both ST2L and SIGIRR were upregulated in RP105^−/−^ mice compared to the WT (Figure 4E and F, p<0.05 for both inhibitors).

### RP105 Modulates Monocyte Inflammatory Response

To study the effects of RP105 deficiency on the inflammatory status of monocytes, whole blood from RP105^−/−^ and WT mice was incubated with LPS or PBS as control. The expression of the Ly6C antigen was used to differentiate between “pro-inflammatory” Ly6C^hi^ and “repair-associated” Ly6C^l^° monocyte subtypes. Total monocyte and monocyte subtype (Ly6C^hi^ and Ly6C^l^°) numbers were similar between RP105^−/−^ and WT mice (Figure 4G) and LPS stimulation in vitro had no effects on the fraction of monocytes subtypes (Figure 4H and I).

The mean fluorescence intensity (MFI) of CD11b was used as monocyte activation marker. Monocyte baseline CD11b expression was equal between the two mouse strains; however, LPS stimulation resulted in increased MFI of CD11b in monocytes from RP105^−/−^ mice compared to WT mice (Figure 4J, p = 0.01), indicating increased TLR4-mediated monocyte activation in blood from R105^−/−^ mice compared to WT mice. More specifically, the difference in monocyte activation state was the result of the Ly6C^hi^ subtype alone. At baseline, the pro-inflammatory Ly6C^hi^ monocyte subtype of RP105^−/−^ mice already showed an increased MFI of CD11b, which increased even further after LPS stimulation compared to Ly6C^hi^ monocytes from WT mice (Figure 4K). The MFI of CD11b on Ly6C^l^° monocytes did not differ between RP105^−/−^ and WT mice (Figure 4L).

### RP105 Deficiency Alters Inflammatory Monocyte Infiltration

**Figure 5:**
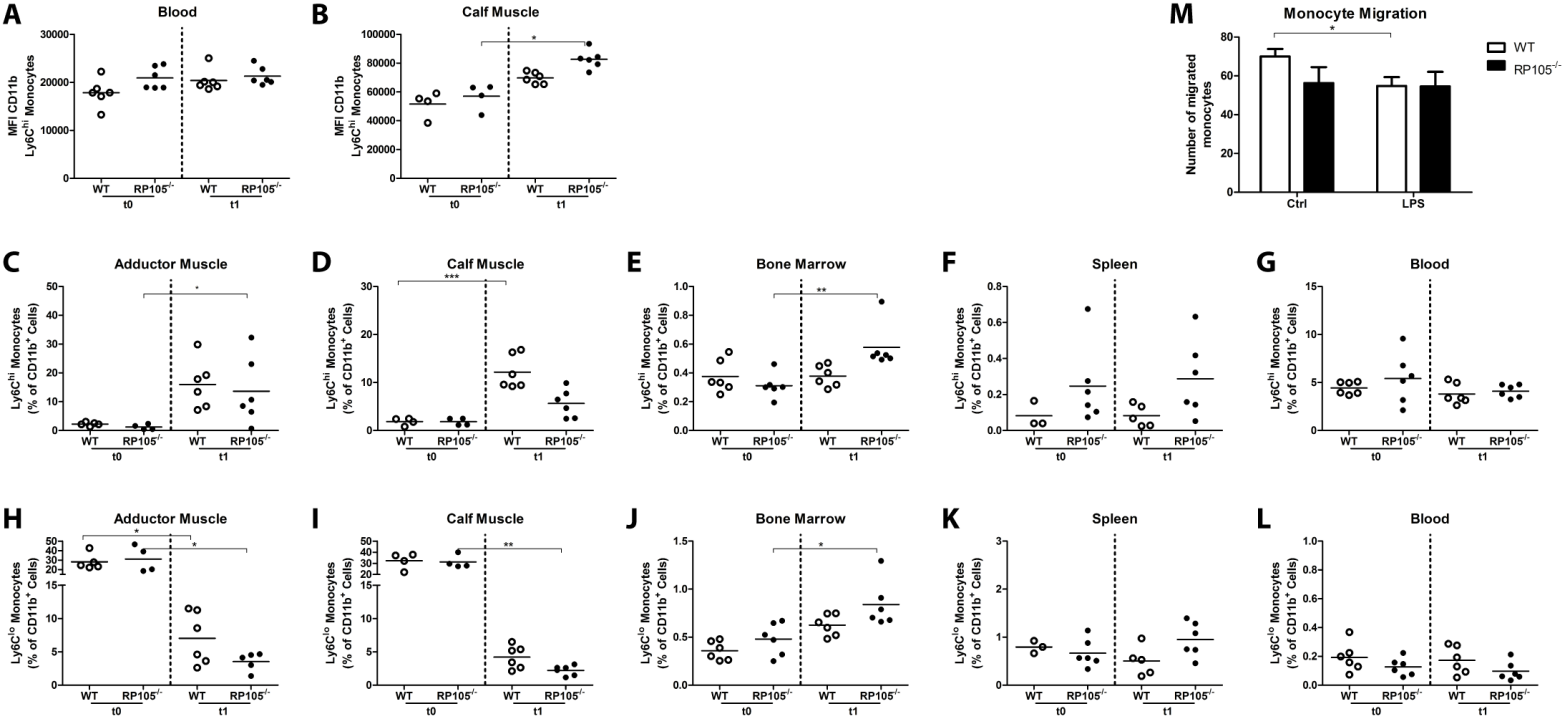
Recruitment of Ly6C^hi^ monocytes in RP105^−/−^ mice in vivo. Flow cytometry analysis of monocytes subtypes (Ly6C^hi^ and Ly6^l^°) before (t0) and 1 day after (t1) HLI in WT and RP105^−/−^ mice (n = 6 WT t0; n = 6 WT t1; n = 6 RP105^−/−^ t0; n = 6 RP105^−/−^ t1). Activation state of Ly6C^hi^ monocytes measured by mean fluorescence intensity (MFI) of CD11b in blood (**A**) and ischemic gastrocnemius muscle (**B**). Flow cytometry analysis of the Ly6C^hi^ monocyte population in adductor muscle group (**C**), gastrocnemius muscle (**D**), bone marrow (**E**), spleen (**F**) and blood (**G**). Flow cytometry analysis of the Ly6C^l^° monocyte population in adductor muscle group (**H**), gastrocnemius muscle (**I**), bone marrow (**J**), spleen (**K**) and blood (**L**). **M**, WT and RP105^−/−^ bone marrow-derived monocyte migration with and without LPS stimulation (10 ng/ml) (n = 4). *P<0.05, **P<0.01, ***P<0.001.

Next, we evaluated Ly6C^hi^ monocyte activation in vivo. Spleen, bone marrow, blood, ipsilateral adductor and gastrocnemius muscle of RP105^−/−^ and WT mice were harvested before (t0) and 1 day (t1) after induction of HLI and were analyzed by FACS. Circulating Ly6C^hi^ monocytes in RP105^−/−^ mice appeared to show higher baseline CD11b expression, indicating a higher activation status, compared to WT mice (Figure 5A), but the induction of HLI did not result in additional monocyte activation in the blood of RP105^−/−^ mice. Interestingly, whereas the baseline activation states of monocytes in the ischemic gastrocnemius muscle were similar in both strains, the MFI of CD11b in RP105^−/−^ mice was significantly increased after HLI compared to baseline (Figure 5B), suggesting increased monocyte activation.

Since circulatory Ly6C^hi^ monocytes in RP105^−/−^ mice showed an activated phenotype already at baseline, we evaluated the migratory abilities of circulating Ly6C^hi^ monocytes in response to HLI. Ly6C^hi^ monocytes were recruited to the adductor and gastrocnemius muscle of the ligated limb in both mouse strains (Figure 5C–D). In RP105^−/−^ mice however, the recruitment to the ischemic gastrocnemius muscle was impaired compared to the WT. At day 1 after HLI, the infiltrated Ly6C^hi^ population in the ischemic gastrocnemius muscles of RP105^−/−^ mice was reduced by 54% compared to WT mice. Interestingly, in the bone marrow of RP105^−/−^ mice, post-ischemic Ly6C^hi^ monocytes increased relative to baseline (Figure 5E). Also, Ly6C^hi^ monocytes appeared higher in the spleen of RP105^−/−^ mice compared to WT mice (Figure 5F), suggesting retention of Ly6C^hi^ monocytes in the spleen and bone marrow of RP105^−/−^ mice. The number of circulatory Ly6C^hi^ monocytes however was not affected by induction of HLI in either strain (Figure 5G).

The aforementioned increase of Ly6C^hi^ monocytes in the post-ischemic adductor and gastrocnemius muscle, albeit less in RP105^−/−^ mice compared to WT mice, was accompanied by a decrease in the number of Ly6C^l^° monocytes after HLI in both mouse strains (Figure 5H–I). Like the Ly6C^hi^ monocyte population, the Ly6C^l^° population was increased in bone marrow of RP105^−/−^ mice compared to WT mice (Figure 5J–K). The number of circulatory Ly6C^l^° monocytes was not affected by inducing HLI in either strain (Figure 5L).

### RP105 Deficiency Alters Migratory Properties of Monocytes

In vitro migration assays on WT monocytes showed that the migratory capacity of monocytes decreased after activation with LPS. In RP105^−/−^ monocytes, activation with LPS could not further reduce monocyte migration (Figure 5M). In correspondence, expression of Cathepsin S (CTSS) was higher in WT monocytes than in RP105^−/−^ monocytes (Figure S2A, p<0.001). Furthermore, PI3K and Rac1 appeared upregulated in WT but not RP105^−/−^ monocytes after LPS stimulation (Figure S2B and C, p = 0.07 for both PI3K and Rac1). MMP2 levels were higher in RP105^−/−^ monocytes however, whereas MMP9 levels were similar between monocytes from both mouse strains (Figure S2D and E). These effects were not observed in whole bone marrow (Figure S2F), implicating that they are monocyte-specific.

### LPS Activation in Hind Limb Ischemia Model

**Figure 6:**
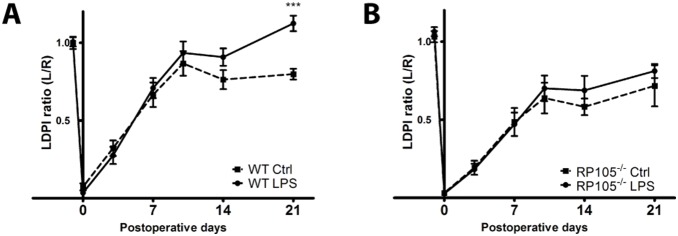
Monocyte activation in RP105^−/−^ mice after induction of HLI. LDPI quantification of WT (**A**) and RP105^−/−^ (**B**) mice. Mice were injected with LPS (1 µg/mouse) in PBS (n = 11 WT; n = 11 RP105^−/−^) or PBS alone (n = 9 WT; n = 9 RP105^−/−^) at 3 days after induction of HLI. Data are calculated as the ratio of ligated over non-ligated paw. All values are presented as the mean ± SEM. Ctrl = control. ***P<0.001.

To further evaluate the effects of the differences in monocyte activation between the two strains of mice in vivo, LPS (1 µg) was injected (i.p) in both RP105^−/−^ and WT mice 3 days after induction of HLI. In correspondence with previous studies [16], we observed that blood flow recovery was increased after LPS injection in WT mice (Figure 6A, day 21 p<0.001). However, no increases in blood flow recovery were observed in RP105^−/−^ mice injected with LPS (Figure 6B), indicating that additional monocyte activation did not occur in RP105^−/−^ mice.

## Discussion

We demonstrate here that the TLR4 accessory molecule RP105 plays an important role in collateral artery remodeling. RP105 deficiency results in enhanced inflammation and increased activation of “pro-inflammatory” Ly6C^hi^ monocytes. However, blood flow recovery after induction of HLI is strongly impaired in RP105^−/−^ mice. This is accompanied by a reduced infiltration of Ly6C^hi^ monocyte subpopulation into the affected muscle tissues. Following induction of HLI, “pro-inflammatory” Ly6C^hi^ monocyte populations accumulated in monocyte storage reservoirs (bone marrow and spleen) in RP105^−/−^ mice, instead of migrating to the affected muscle tissues of the ischemic hind limb. As monocytes are among the first cells to infiltrate the affected tissues and contribute to the initiation and proliferation of the arteriole remodeling process [2], impaired monocyte recruitment hampers blood flow recovery even in the later stages of vascular remodeling. Our data indicate that activation of pro-inflammatory monocytes must occur in the proper compartment to contribute effectively to the pro-arteriogenic response.

Following HLI, we observed a marked increase of both TLR4 and RP105 mRNA expression in adductor and gastrocnemius muscles of WT C57BL/6 mice, a mouse strain displaying effective arteriogenesis [43]. A joint upregulation of TLR4 and RP105 had previously also been described in both human and mouse macrophages, where RP105 directly mimics expression of TLR4 [32]. Although RP105 is expressed on several different cell types, in our mouse models, RP105^+^ cells all stained positive for MOMA-2 and were found in the perivascular space of remodeling collateral arteries, clearly indicating a role for RP105^+^ monocytes and/or macrophages in arteriogenesis.

RP105 was described as a negative regulator of TLR4 response, resulting in increased pro-inflammatory NF-κB-mediated gene transcription in RP105^−/−^ mice [31], [32]. Previous reports showed that LPS, via TLR4, increases arteriogenesis [16] and that TLR4 deficiency hampers blood flow recovery after HLI [12]. Therefore, we originally hypothesized that arteriogenesis would be enhanced in RP105^−/−^ mice, as TLR4-mediated inflammation is critical for effective arteriogenesis. Unexpectedly, RP105^−/−^ mice showed a reduced blood flow recovery after HLI compared to WT mice. This functional impairment in RP105^−/−^ mice was confirmed by reduced expansive remodeling of αSMA^+^ vessels in the central part of the adductor muscle group.

Since no differences in pre-existing collateral densities were observed and the drop in blood flow directly after induction of HLI was similar in RP105^−/−^ and WT mice, we conclude that the reduced blood flow recovery in RP105^−/−^ mice after HLI was the result of reduced arteriogenesis alone.

The LPS-induced inflammatory response was increased in RP105^−/−^ mice, both in vivo and ex vivo. As blood flow recovery following HLI was decreased in RP105^−/−^ mice, we hypothesized that the increased activation of monocytes in these mice is inappropriate. It has been established that monocytes drive arteriogenesis and that they express high levels of both TLR4 and RP105. TLR4 is crucial in the activation of monocytes. TLR4 expression levels are higher on monocytes from patients with peripheral [44] or coronary artery disease [45] than in healthy individuals. Since the “pro-inflammatory” Ly6C^hi^ monocytes are more responsive to TLR stimulation and are the predominant TNFα producing monocytes [24], we focused on the activation state of Ly6C^hi^ monocytes in particular.

Compared to WT mice, we indeed observed increased activation of Ly6C^hi^ monocytes in RP105^−/−^ mice ex vivo, both at baseline and after LPS stimulation. Also in vivo, circulatory Ly6C^hi^ monocyte activation was increased at baseline in RP105^−/−^ mice compared to WT mice. Even though TLR inhibitors ST2L and SIGIRR were upregulated in RP105^−/−^ mice compared to the WT, this was not sufficient to dampen the activation state of the RP105^−/−^ monocytes. It cannot be excluded that upregulation of ST2L and SIGIRR has an effect on arteriogenesis however. The alternative splice variant of ST2L, soluble ST2 (sST2), has been implicated in cardiovascular disease [46] and although no such reports exist for SIGIRR, a causative role cannot be excluded.

However, unlike in WT mice, the circulatory Ly6C^hi^ monocyte population of RP105^−/−^ could not be further activated by induction of HLI. In the ischemic gastrocnemius muscle however, we did observe an increase in Ly6C^hi^ monocyte activation in both strains, a response that was exaggerated in the RP105^−/−^ mice. However, even though the activation state of Ly6C^hi^ monocytes was increased in RP105^−/−^ mice, their numbers in the affected tissues were not. A previous study on the role of TLRs in arteriogenesis showed that even very subtle decreases in monocyte numbers around remodeling collaterals can have detrimental effects [12]. In WT mice, we observed increases in Ly6C^hi^ monocyte numbers in the affected adductor and gastrocnemius muscles in the initial phase of arteriogenesis. In the affected muscles of RP105^−/−^ mice, this response was impaired. Recent reports describe that Ly6C^hi^ monocytes accumulate in ischemic myocardium after myocardial infarction [47] and are recruited to ischemic muscles in the early stages after ischemia [48], [49]. These reports showed the potential of the Ly6C^hi^ subtype to regulate post-ischemic vessel growth in adoptive transfer studies, whereas adoptive transfer of the Ly6C^l^° subtype did not contribute to post-ischemic revascularization.

In contrast, Ly6C^hi^ monocytes accumulated in the bone marrow and spleen of RP105^−/−^ mice after induction of HLI. The bone marrow is a reservoir for Ly6C^hi^ monocytes [48] and also the spleen stores monocytes, which are rapidly deployed after, for example, the induction of myocardial infarction in mice [47]. The retention of Ly6C^hi^ monocytes in the bone marrow and spleen of RP105^−/−^ mice suggests that, when normally expressed, RP105 enables recruitment and migration of these monocytes from their storage compartments to the affected adductor and gastrocnemius muscles. In vitro monocyte migration assays and expression analyses of migratory molecules support this role of RP105.

We believe that the increased activation state of Ly6C^hi^ monocytes in RP105^−/−^ mice at baseline and following HLI compared to WT mice is inappropriate and negatively affects their migratory abilities and thus hampers the regenerative response of RP105^−/−^ mice after ischemia.

Before HLI, the predominant resident monocyte subtype was the Ly6C^l^° monocyte, making up almost one third of total CD11b^+^ cells in adductor and gastrocnemius muscles of both WT and RP105^−/−^ mice. Following HLI, Ly6C^hi^ monocyte levels increased rapidly in the affected adductor and gastrocnemius muscle of WT mice, accompanied by a rapid decrease of Ly6C^l^° monocytes. It is still unclear whether Ly6C^hi^ monocytes differentiate into Ly6C^l^° monocytes and vice versa inside injured tissue [50] after the initial inflammatory response or whether they are distinct subpopulations recruited separately from their storage compartments [51]. In ex vivo whole blood stimulation assays, where migration is eliminated as a contributing factor, we found no differences in the numbers of these monocyte subtypes after stimulation with LPS in either mouse strain, suggesting that the differences in monocyte subtype numbers observed after HLI are indeed caused by differences in monocyte migration and not monocyte differentiation.

In conclusion, the TLR4 accessory molecule RP105 is expressed by monocytes/macrophages in areas of expansive collateral artery remodeling and plays an important role in blood flow recovery after hind limb ischemia. RP105 deficiency results in an unrestrained inflammatory response and monocyte over-activation, most likely due to the lack of TLR4 regulation. Inappropriate, premature systemic activation of monocytes, more specifically of the “pro-inflammatory” Ly6C^hi^ monocyte subtype, results in reduced infiltration of Ly6C^hi^ monocytes in ischemic tissue and consequently in reduced arteriogenesis and blood flow recovery.

## Supporting Information

Figure S1 **(A)** Lumen area (µm^2^) of αSMA+ vessels per section and fraction **(B)** of large αSMA^+^ vessels (>200 µm), measured at the center of the adductor muscle group in ligated and non-ligated limbs of WT (n = 6) and RP105^−/−^ (n = 6) mice. **(C)** Capillary area of CD31^+^ vessels per section (%), size index **(D)** and number index **(E)** in ligated and non-ligated limbs of WT (n = 6) and RP105^−/−^ (n = 6) mice, measured in the gastrocnemius muscles. ns = non-significant. All values are presented as the mean ± SEM. *P<0.05, **P<0.01, ***P<0.001.
(TIF)

Figure S2 **Bone marrow derived monocytes (10^6^) were incubated with LPS (10 ng/ml) or control medium (RPMI) overnight, after which the cells were lysed in Trizol and total RNA was extracted.** Monocyte mRNA expression of CTSS **(A)**, PI3K **(B)**, Rac1 **(C)**, MMP2 **(D)** and MMP9 **(E)** was measured by real-time quantitative PCR (n = 4). **(F)** Whole bone marrow was lysed in Trizol and total RNA was extracted. mRNA expression levels of CTSS, PI3K, RAC1, MMP2 and MMP9 were measured by real-time quantitative PCR (n = 4). *P<0.05, **P<0.01, ***P<0.001.
(TIF)

Figure S3 **Gating strategy for peripheral blood monocyte subpopulations.** A gate is drawn on all cells in a FCS/SSC plot (A) to exclude debris. Of the cells gated in plot A, the expression of CD11b (X-axis) and B220 (Y-axis) is shown in plot B, on which gates are placed on the CD11bneg cells and the CD11pos cells. The CD11bneg cells gated in plot B showing expression of the B-cell marker B220 (X-axis) and Ly6C (Y-axis): B220neg/Ly6Cneg cells represent the T-cells, B220pos/Ly6Cneg cells are B-cells, B220neg/Ly6Cpos cells are activated T-cells and B220pos/Ly6Cpos cells are plasmacytoid dendritic cells (pDCs). The CD11pos cells from plot B are selected in plot D showing expression of Ly6G (X-axis) and SSC (Y-axis), in which Ly6Gpos/SSChi cells represent neutrophilic granulocytes, Ly6Gneg/SSChi cells represent eosiniphilic granulocytes and the Ly6Gneg/SSClo cells represent the non-granulocytic cells. These latter cells are selected in plot E, showing expression of CD11b (X-axis) and CD115 (Y-axis): CD11bhi/CD115hi cells represent the monocytes and the CD11bdim/CD115neg cells represent NK cells. The monocytes gated in plot E are selected in plot F, showing their expression of CD115 (X-axis) and Ly6C (Y-axis): Ly6Chi cells represent the pro-inflammatory monocytes, Ly6Cmed cells represent the intermediate monocyte population and Ly6Clo cells represent the anti-inflammatory, pro-angiogenic/repair-associated monocytes. Figure G shows a summary of the hierarchy of the characterized cell populations.
(TIF)
