# Supplementary figures and images for: TLR4 Accessory Molecule RP105 (CD180) Regulates Monocyte-Driven Arteriogenesis in a Murine Hind Limb Ischemia Model
Source: PLoS One. 2014 Jun 19;9(6):e99882. doi: 10.1371/journal.pone.0099882 (PMC4063870; doi:10.1371/journal.pone.0099882)

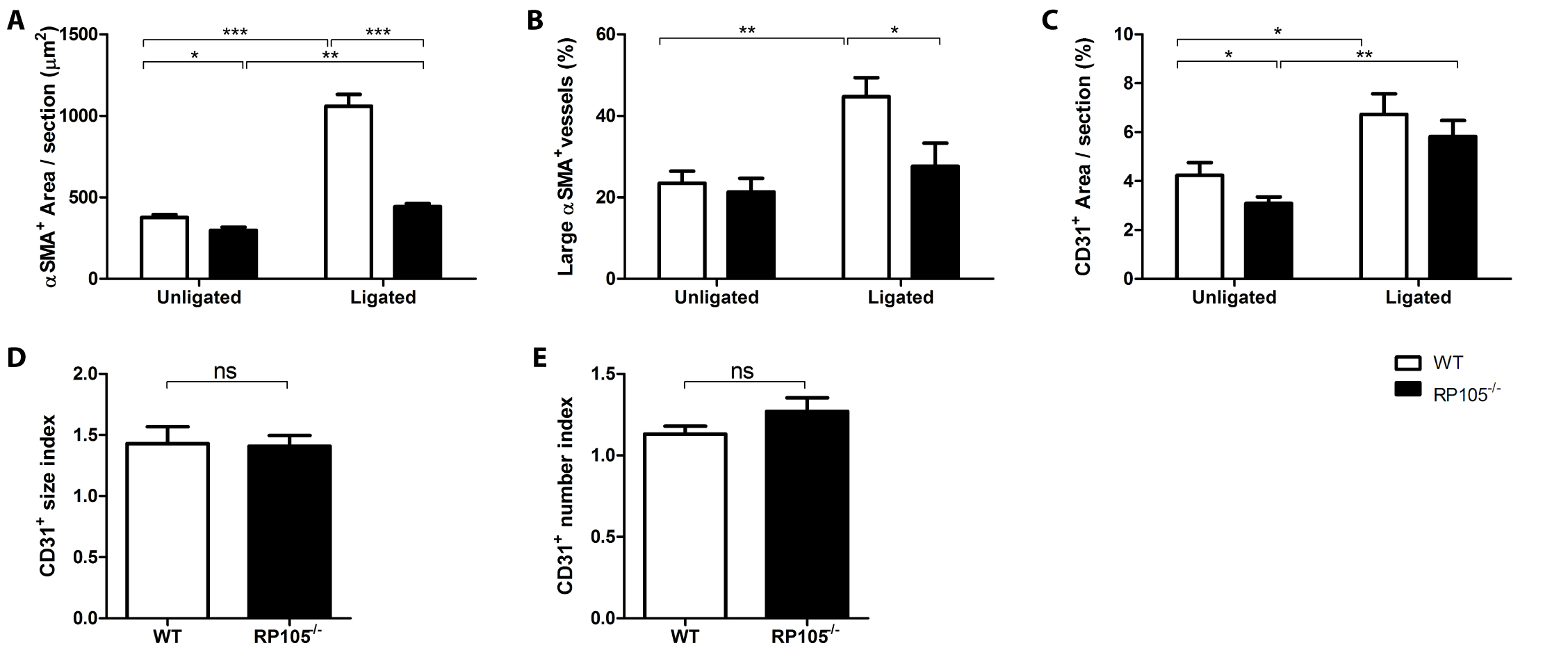

Supplement: Figure S1 — (A) Lumen area (µm2) of αSMA+ vessels per section and fraction (B) of large αSMA+ vessels (>200 µm), measured at the center of the adductor muscle group in ligated and non-ligated limbs of WT (n = 6) and RP105−/− (n = 6) mice. (C) Capillary area of CD31+ vessels per section (%), size index (D) and number index (E) in ligated and non-ligated limbs of WT (n = 6) and RP105−/− (n = 6) mice, measured in the gastrocnemius muscles. ns = non-significant. All values are presented as the mean ± SEM. *P<0.05, **P<0.01, ***P<0.001. (TIF) [file pone.0099882.s001.tif]

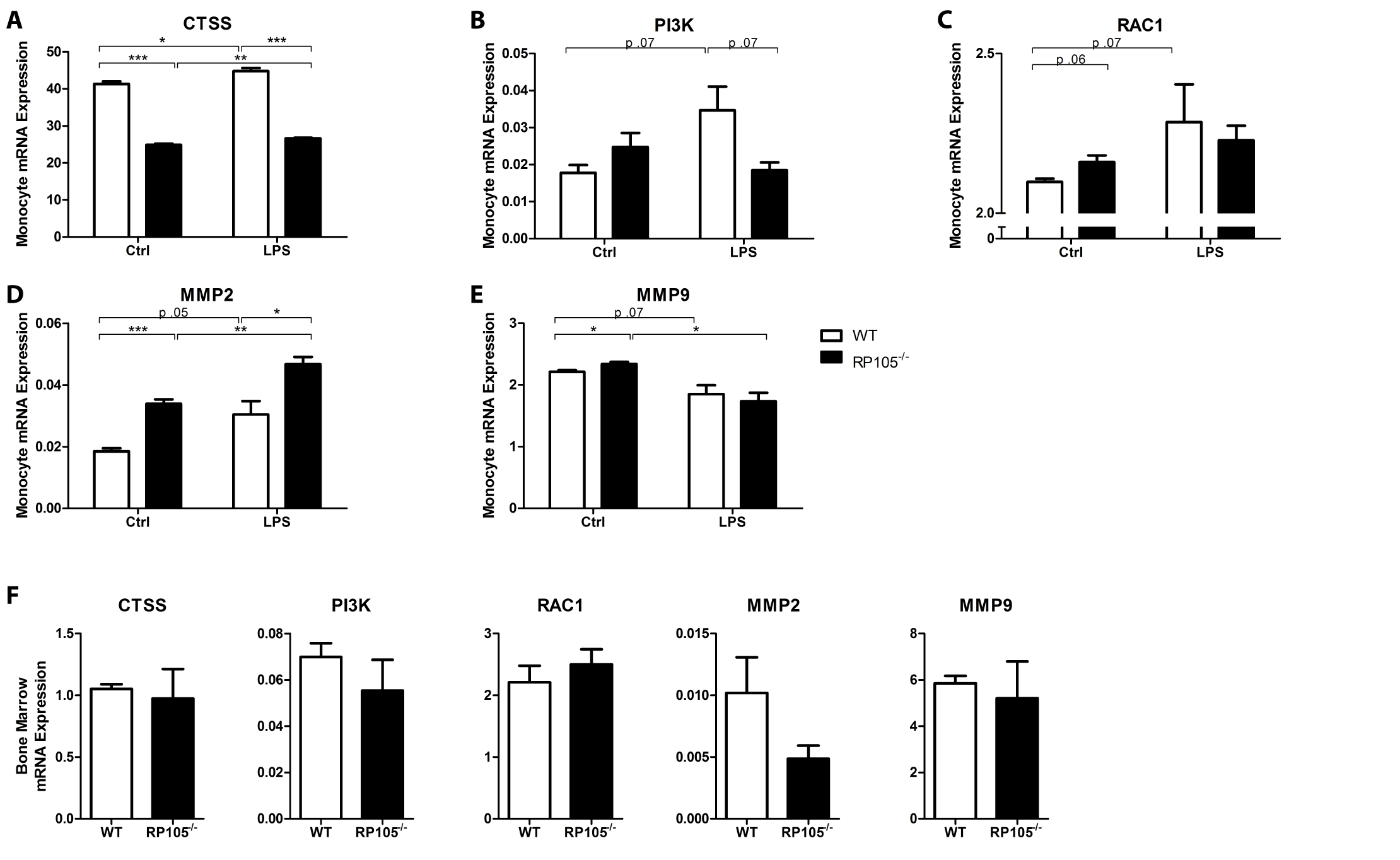

Supplement: Figure S2 — Bone marrow derived monocytes (106) were incubated with LPS (10 ng/ml) or control medium (RPMI) overnight, after which the cells were lysed in Trizol and total RNA was extracted. Monocyte mRNA expression of CTSS (A), PI3K (B), Rac1 (C), MMP2 (D) and MMP9 (E) was measured by real-time quantitative PCR (n = 4). (F) Whole bone marrow was lysed in Trizol and total RNA was extracted. mRNA expression levels of CTSS, PI3K, RAC1, MMP2 and MMP9 were measured by real-time quantitative PCR (n = 4). *P<0.05, **P<0.01, ***P<0.001. (TIF) [file pone.0099882.s002.tif]

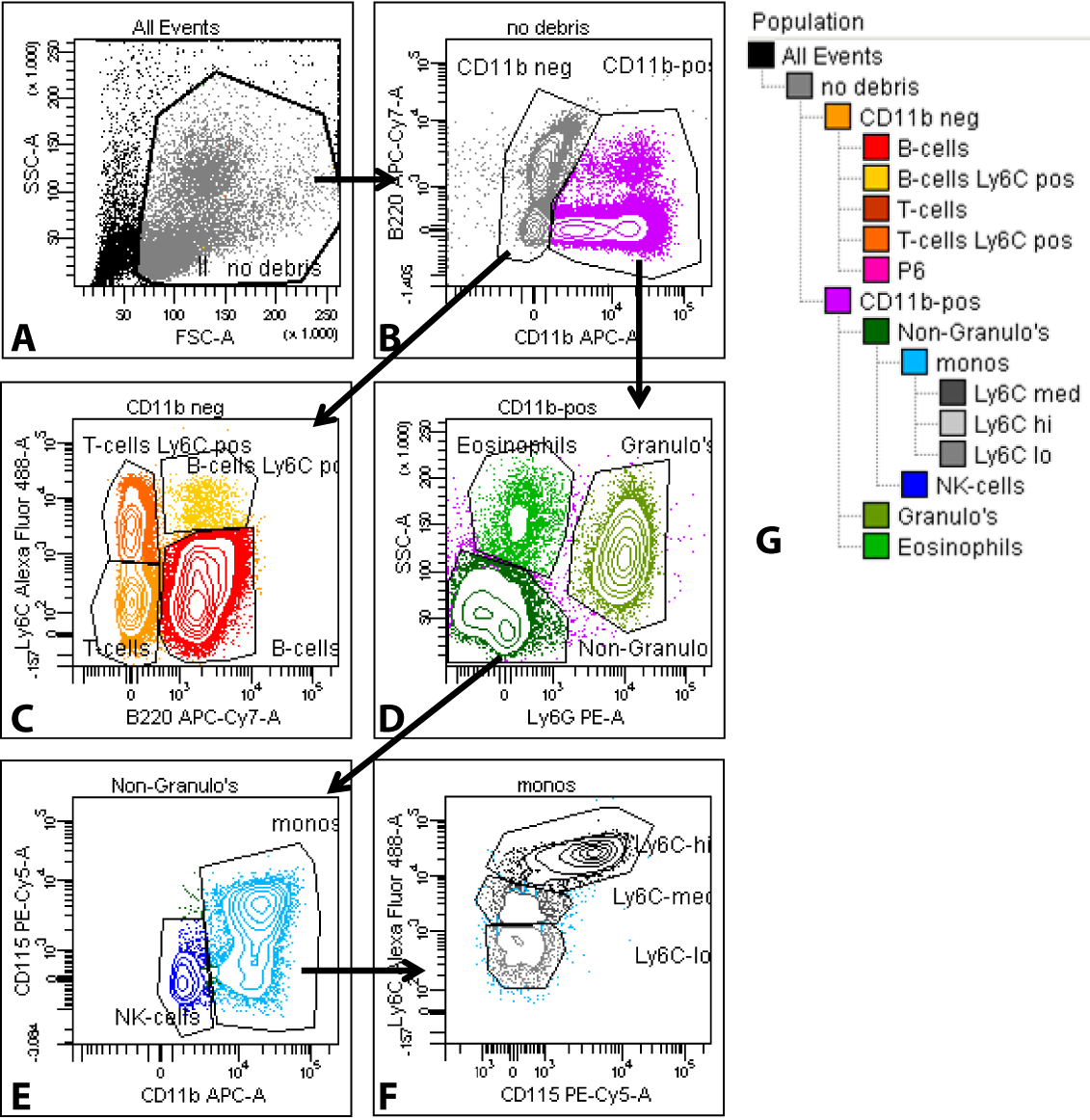

Supplement: Figure S3 — Gating strategy for peripheral blood monocyte subpopulations. A gate is drawn on all cells in a FCS/SSC plot (A) to exclude debris. Of the cells gated in plot A, the expression of CD11b (X-axis) and B220 (Y-axis) is shown in plot B, on which gates are placed on the CD11bneg cells and the CD11pos cells. The CD11bneg cells gated in plot B showing expression of the B-cell marker B220 (X-axis) and Ly6C (Y-axis): B220neg/Ly6Cneg cells represent the T-cells, B220pos/Ly6Cneg cells are B-cells, B220neg/Ly6Cpos cells are activated T-cells and B220pos/Ly6Cpos cells are plasmacytoid dendritic cells (pDCs). The CD11pos cells from plot B are selected in plot D showing expression of Ly6G (X-axis) and SSC (Y-axis), in which Ly6Gpos/SSChi cells represent neutrophilic granulocytes, Ly6Gneg/SSChi cells represent eosiniphilic granulocytes and the Ly6Gneg/SSClo cells represent the non-granulocytic cells. These latter cells are selected in plot E, showing expression of CD11b (X-axis) and CD115 (Y-axis): CD11bhi/CD115hi cells represent the monocytes and the CD11bdim/CD115neg cells represent NK cells. The monocytes gated in plot E are selected in plot F, showing their expression of CD115 (X-axis) and Ly6C (Y-axis): Ly6Chi cells represent the pro-inflammatory monocytes, Ly6Cmed cells represent the intermediate monocyte population and Ly6Clo cells represent the anti-inflammatory, pro-angiogenic/repair-associated monocytes. Figure G shows a summary of the hierarchy of the characterized cell populations. (TIF) [file pone.0099882.s003.tif]
